# Supplementary material for: Using the ADAPT guidance to culturally adapt a brief intervention to reduce alcohol use among injury patients in Tanzania
Source: PLOS Glob Public Health. 2025 Feb 6;5(2):e0004200. doi: 10.1371/journal.pgph.0004200 (PMC11801724; doi:10.1371/journal.pgph.0004200)

Table of reporting guidelines from:

O’Brien, Bridget C., PhD; Harris, Ilene B., PhD; Beckman, Thomas J., MD; Reed, Darcy A., MD, MPH; Cook, David A., MD, MHPE Standards for Reporting Qualitative Research, Academic Medicine: September 2014 - Volume 89 - Issue 9 - p 1245-1251

doi: 10.1097/ACM.0000000000000388


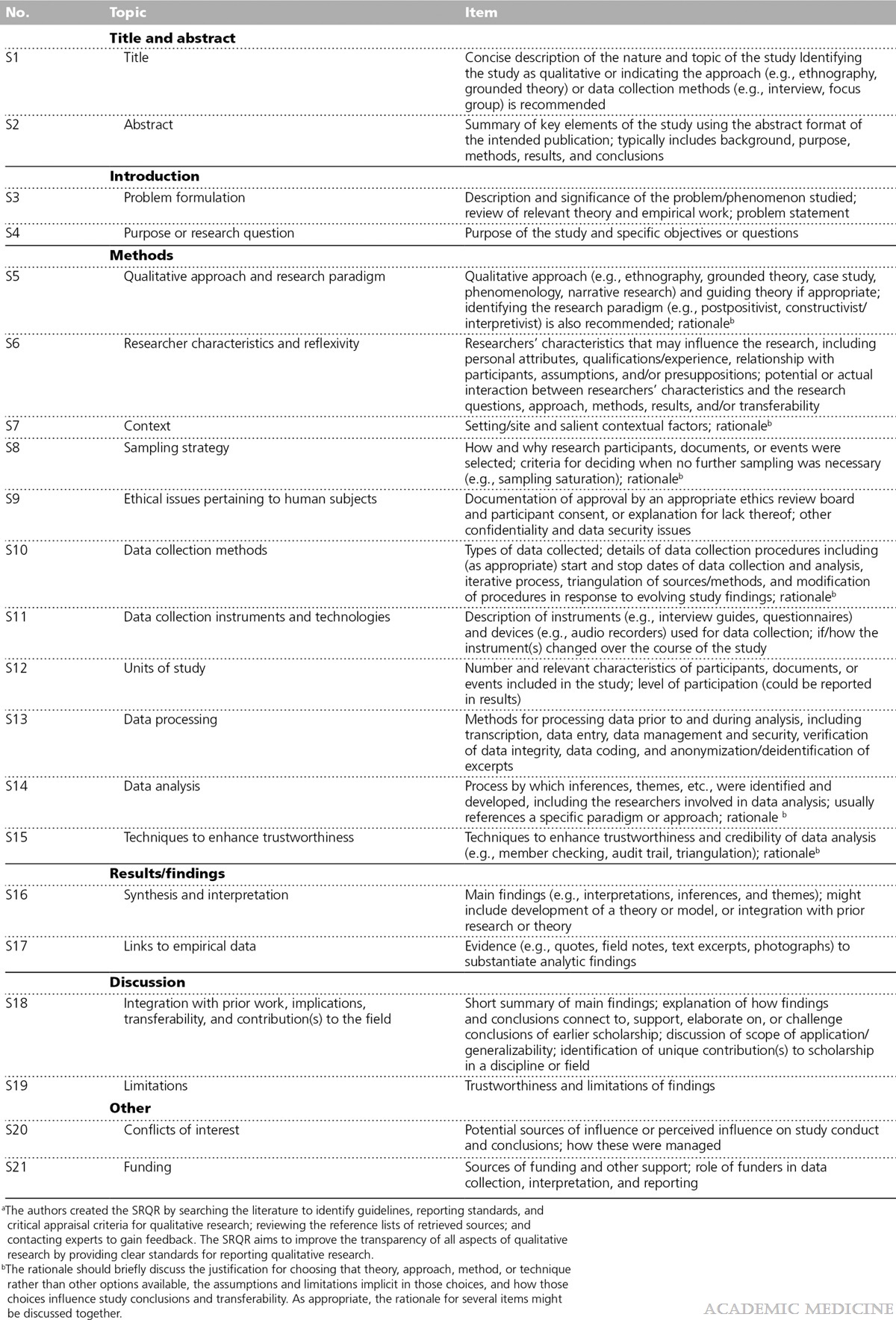

Supplement: S1 Text — (DOCX) [file pgph.0004200.s002.docx]
