# Supplementary material for: Using the ADAPT guidance to culturally adapt a brief intervention to reduce alcohol use among injury patients in Tanzania
Source: PLOS Glob Public Health. 2025 Feb 6;5(2):e0004200. doi: 10.1371/journal.pgph.0004200 (PMC11801724; doi:10.1371/journal.pgph.0004200)
Supplement: Attachment — Submitted filename: Rebutal letter.docx [file pgph.0004200.s003.docx]

Dear Editors,

I am writing to resubmit our research paper titled "Using the ADAPT Guidance to Culturally Adapt a Brief Intervention for Reducing Alcohol Use Among Injury Patients in Tanzania" for your consideration in PLOS Global Public Health.

In response to the feedback received from the editor regarding the omission of patient enrollment dates in our previous submission, we have revised the manuscript to include this critical information. Additionally, we have attached ethical approval documents from the Tanzanian National Institute for Medical Research (NIMR), Kilimanjaro Christian Medical University College (KCMUCo), Kilimanjaro Christian Medical Centre (KCMC) and Duke University to confirm the study's authorization.

As a clarification, Drs. Mvungi, Ngocho, and Njau have been included on this project for regulatory purposes due to roles they played in this multi-part protocol's early years. In the case of the present manuscript, none were involved or meet authorship criteria.
