# Supplementary material for: Using the ADAPT guidance to culturally adapt a brief intervention to reduce alcohol use among injury patients in Tanzania
Source: PLOS Glob Public Health. 2025 Feb 6;5(2):e0004200. doi: 10.1371/journal.pgph.0004200 (PMC11801724; doi:10.1371/journal.pgph.0004200)
Supplement: Attachment — Submitted filename: Response to reviwers.docx [file pgph.0004200.s004.docx]

**Reviewer #1: Review Reports**
Title: Using the ADAPT guidance to culturally adapt a brief intervention to reduce alcohol use among injury patients in Tanzania
Review Comments

**A. Is that adaption or adoption?If so what are adopted?What are not?What are adapted?what are not?**

Answer: The current manuscript references cultural adaptation of the intervention. Details of elements of previously existing interventions which were modified, eliminated, or added to suit the local context are included in methods and results sections.

**B.Is that tool adaption or intention to use the guideline, make sure to state it.**

Answer: The current paper employs the ADAPT guidance as a framework for addressing the adaptation of the intervention to a novel setting, using the ADAPT. This is noted in the methods section.

**C.The abstract should be rewritten e.g. The methods iss incomplete and the result contains methods.**

Answer: We have made changes to the methods and results section in the abstract to reflect both the cultural adaptation and the quantitative evaluation.

**D.On the background**
-It's well written

Answer: Thank you.

**-What intervention was given before the ADAPT guideline?What is the limitations of the guideline? Why the ADAPT guideline is selected? What is is the importance and uniqueness of it?**

Answer: Thank you for your comment. We have updated the "ADAPT guidance" section within the Methods to provide more detail on our rationale for this choice. To address your concerns about the limitations, we have also revised the "Cultural adaptation process" section in the Discussion to more clearly discuss some of the challenges and limitations associated with using the ADAPT guidelines.

**E. On the Methods section
-Lacks logical flow
-Mis use of tenses ' we will'**

Answer: We have revised the document to reduce the use of future tense where appropriate.

**-Unnecessary underlining**

Answer: Thank you for your attention to detail. We have used underlining to distinguish different sub-steps within each step of the ADAPT guideline throughout the manuscript.

**-Niot used references for '...for prior mixed evaluation...'**
Answer: We have added citations to our previous work in that sentence.

**-The validity and reliability is not presented**
Answer: The current manuscript presents adaptation of an intervention. As such, we did not evaluate validity or reliability. But we do report evidence of content contextual validity and translation consistency.

**-The trustworthiness for qualitative and data quality for quantitative data was not presented.**
Answer: See details regarding consensus building and project team membership in ADAPT Step 2, which illustrates an element of qualitative data trustworthiness. A note was added in the methods section for ADAPT Step 3 regarding quantitative data quality.

**-Not reported how the triangulation was done.**

Answer: All sources of data were considered and consensus was sought in cases of conflict. See details under the heading “Areas for adaptation identified and adaptations developed by consensus.”

**F. On the Result and the consequent section**
**-The systematic review is missed and the meta analysis is not presented.**

Answer: The detailed methods and comprehensive results of our systematic review are published in a separate paper, referenced in the manuscript as citation 19. We believe this approach allows us to focus the current manuscript on the adaptation and implementation of the BNI in the Tanzanian context, while still providing readers access to the full systematic review details elsewhere.  **-Tables are not self explanatory**

Answer: We have changed Table titles across the manuscript for more clarity. **-Should be clearly and briefly presented
-The discussion lacks strength for theoretically wel as practilical implications.**

Answer:

**-Lacks some components E.g. funding, conflict of interests**

Answer: We are thankful for the reviewers attention to this matter. The authors submitted disclosures of funding and conflicts of interest through the editorial manager system from PGPH as per the journal instructions.

**Reviewer #2: The article is written clearly and objectively.**
The introduction and methods are well-defined, however, the authors could improve the clarity of the article's objective. It seems to be confused with the objectives of the research as a whole.
The best-described objective would be: "to describe the process of culturally adapting a BNI to reduce post-injury alcohol use for implementation in this patient population."

Answer: We are thankful for your suggestions and have incorporated the changes in our objective.
